# Effects of psychological capital and social support availability on anxiety and depression among Chinese emergency physicians: Testing moderated mediation model

**DOI:** 10.3389/fpsyg.2022.991239

**Published:** 2022-12-07

**Authors:** Haibo Xu, Lixin Peng, Zhen Wang, Xin Liu

**Affiliations:** ^1^Center for Mental Health Education and Research, Xuzhou Medical University, Xuzhou, China; ^2^School of Management, Xuzhou Medical University, Xuzhou, China

**Keywords:** depression, anxiety, psychological capital, social support availability, emergency physician, moderated mediation model

## Abstract

**Introduction:**

Anxiety often precedes depression, and the pathway from anxiety to depression may be affected by multiple exposures. Our research aims to explore the mediating effect of the social support availability (SSA) between anxiety and depression and how it is moderated by psychological capital.

**Methods:**

A cross-sectional study was conducted among Chinese emergency physicians at the top-level general hospitals in eastern China. Data were collected via the questionnaire including anxiety and depression subscales of Symptom Checklist-90, Psychological Capital Questionnaire as well as Social Support Rating Scale. The PROCESS v3.4 macro was employed to assess the mediating role of SSA and a moderating role of psychological capital.

**Results:**

A total of 536 valid samples were filtered. Anxiety, depression, SSA, and psychological capital were significant correlated. Anxiety was positively associated with depression (β = 0.82, *p* < 0.001), and the SSA mediated the relationship between anxiety and depression (indirect effect = 0.013, 95%BootCI [0.005, 0.023]). Psychological capital (specifically, self-efficacy, hope and resilience) further played a moderating role in the relationship between SSA and depression (β = 0.06, *p* < 0.01).

**Conclusion:**

The mental health of emergency physicians should be concerned. In order to decrease anxiety and depression, SSA and psychological capital should be increased as the interventions for emergency physicians.

## Introduction

Depression is common in medical practitioners across all stages of their careers (Center et al., 2003; Bailey et al., 2018), even occurs much more frequently than in the general population (Eckleberry-Hunt and Lick, 2015). Medical staffs always have poorer mental health than the general population (Rajasekar and Krishnan, 2021). The morbidity of depression among medical staff was estimated between 12.3 and 45.5% (Paiva et al., 2018; de Wit, 2020; Elhadi et al., 2020). In addition, anxiety is also the common mental health problem for medical staff (Medisauskaite and Kamau, 2019). A Brazilian survey showed that the positive rate of anxiety was 56.6% among 1,419 doctors (de Mélo Silva Júnior et al., 2022). Because of the special nature of the work, emergency physicians (EPs) need to be ready to provide medical services for emergency cases and suffer from huge occupational stress (Basu et al., 2020; Janicki et al., 2020; Pujo et al., 2021). A longitudinal study found the anxiety level of medical residents among EPs were higher than other department doctors (González-Cabrera et al., 2018), and a study at east Africa’s largest public hospital displayed that 40.6% of EPs had the moderate to severe range of depression disorders (Iheanacho et al., 2022). There are lots of factors that affect the mental health of EPs and work environment is one of the factors (Rasmussen et al., 2014). In China, large population leads to a crowded and noisy emergency environment, which is not only bad for the work of EPs, but also reduces patients’ satisfaction (Lin et al., 2013). The recent research discovered that a total of 35.6% of EPs suffered from depression among 15,243 Chinese samples (Chen et al., 2022).

### The relationship between anxiety and depression

Anxiety disorders refer to a group of mental disorders in which patients experience excessive worry and constant fear, and depression is a common psychiatric disorder characterized by sadness, loss of interest or pleasure, feelings of tiredness and poor concentration (World Health Organization, 2017). Because of similarity in symptoms, anxiety and depression often go together (Groen et al., 2020) as the most common comorbidity in psychiatry, while the mechanism of the comorbidity is still unclear (Ter Meulen et al., 2021). Previous study discovered that the overlapping symptoms act as a bridge linking the anxiety and depression (Groen et al., 2020). Nevertheless, either anxiety or depression has a respective component to differentiate both, for anxiety is characterized by psychological hyperarousal and depression is characterized by anhedonia (Joiner, 1993), and anxiety precedes depression in the temporal order of onset of most cases (Moscati et al., 2016; Demyttenaere and Heirman, 2020). Starr et al. (2014) found that anxiety may develop into depression if limiting interpersonal interaction, because the decrease or loss of social relationships is harmful to mental health (Chu, 2017). In conclusion, anxiety and depression are highly correlated and anxiety may be a precursor to depression. Thus, we proposed the hypothesis.

Hypothesis 1: anxiety is highly related to depression among EPs in China.

### The mediating role of social support availability

Social support, as a crucial social factor beneficial to human health, has long been recognized and well-documented for several decades (Cobb, 1976; Ditzen and Heinrichs, 2014), but the concrete definition of social support is not uniform (Cohen, 1988). In general, social support is defined as the material, information and emotional support that an individual receives in a network of social relationships (Cohen and Wills, 1985; Cohen, 1988; Ditzen and Heinrichs, 2014). That means, the individual’s social support concerns two parties: actual received social support and subjective perceived social support. The social support availability (SSA) is defined as the extent to which individuals utilize the social support that available to them, which belongs to subjective perceived social support (Xiao, 1994). Compared with actual received social support, the SSA should be most effective in altering subjective cognitive appraisals if the counter the specific needs elicited by the stressful event (Cohen and McKay, 2020). In term of the relationship between stress or negative emotions and perceived social support, Barrera proposed the social support deterioration model that stress or negative emotions can deteriorate the perceived availability or effectiveness of social support (Barrera, 1986). Hindmarch (1998) found that anxious individuals will have cognitive biases and cannot perceive things correctly, which verified the deterioration model. Existing empirical research found that perceived social support can mediate the relationship between anxiety and life satisfaction (Yu et al., 2020), quality of life (Kugbey et al., 2020; Yang and Lu, 2022), and depression (Dour et al., 2014). However, less research has been done on the relationship between SSA (as a component of social support) and anxiety or depression. A study found that anxiety was negatively associated with perceived availability of social support (Den Oudsten et al., 2010). And a recent survey found social support including the dimension of SSA mediated the relationship between the psychological capital and depression among EPs in China (Xu et al., 2022a). Furthermore, low levels of perceived social support may lead to hopelessness and helplessness, which in turn can trigger depression (Johnson et al., 2001). Nonetheless, the role of SSA in the relationship between anxiety and depression was still unclear. Thus, based on the deterioration model, we proposed this hypothesis.

Hypothesis 2: SSA paly a mediating role in the relationship between anxiety and depression among EPs in China.

### The moderating role of psychological capital

Psychological capital (PsyCap), as an emerging positive psychological resource, is defined as ‘a positive state of mind exhibited during the growth and development of an individual,’ comprising four core components: self-efficacy, optimism, hope, resilience (Luthans et al., 2007b). PsyCap has been proven to be effective in improving individual performance and relieving negative emotions. Empirical research showed that PsyCap was negatively associated with psychological symptoms, such as anxiety (Minglu et al., 2020) and depression (Shen et al., 2014), and could improve nurses’ care competencies (Guo et al., 2021) and quality of life of cancer patients (Guo et al., 2022). And PsyCap also played a mediating role in the relationship between anxiety or depression and other related variables (Rahimnia et al., 2013; Jiang, 2021; Zhao et al., 2021). What’s more, PsyCap could moderate the relationship between perceived stress and negative emotions (Wang et al., 2021b) or anxiety (Yang and Yang, 2022). The study of Xu et al. (2022b) found that college freshmen with high level of PsyCap reduced the more effect of interpersonal sensitivity on depression than that with low PsyCap. However, there was little exploration about the moderating effect of PsyCap in the relationship between anxiety, SSA and depression. Hence, we proposed the following hypothesis.

Hypothesis 3: PsyCap has the moderating effect on one or more paths of the mediating model.

**FIGURE 1:**
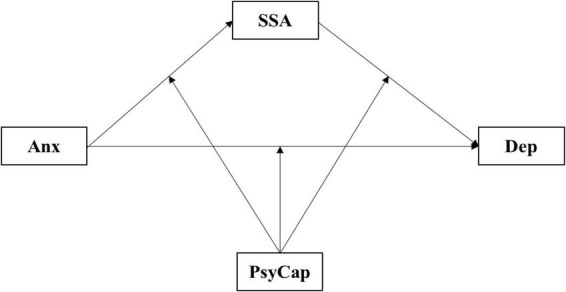
The hypothetical moderated mediation model. Dep, depression; Anx, anxiety; SSA, social support availability; PsyCap, psychological capital.

In summary, based on the appraisal perspective of buffering effect of social support and the dynamic effect model of PsyCap, the present study constructed the moderated mediation model (Figure 1). The aim of our study was to explore the mediating (SSA) and moderating (PsyCap) mechanisms in the relationship between anxiety and depression among EPs in China.

## Materials and methods

### Participants and procedure

This study was performed from July to August 2017 by a cross-sectional survey in Jiangsu Province in eastern China. The dataset was constructed from 33 tertiary grade-A (top-level) general hospitals in 13 prefecture-level cities of the province. Before the formal investigation, researchers first contacted the relevant staffs of hospitals to introduce the purpose and details in order to achieve approval. The researchers recruited college students to train as investigators. After completing the training, the investigators went to the target hospitals that achieved approval with the paper questionnaire to collect data. With the cooperation of the emergency department director, the EPs were called together for on-site questionnaire distribution, filling and retrieval. Before filling out, the investigator told the purpose and guidance of the study. EPs who filled out and submitted questionnaire were considered as contenting to participate in this study.

The inclusion criteria for respondents included: (1) those who volunteered to participate in our survey and followed the principle of informed consent, and (2) who understood the interview questions. The exclusion criteria were: (1) those who refused to sign the informed consent, and (2) the uncompleted answers.

### Measures

An anonymous self-reported questionnaire in Chinese was distributed to all the selected EPs. The questionnaire contained two parts: the first part had the social demographic and other relevant features of the EPs that act as control variables in the model, including gender, age, working-years, education background and professional post; in the second part, we used the following maturity scales as the measuring tool: anxiety and depression subscales of Symptom Checklist 90 (SCL-90), Psychological Capital Questionnaire 24 (PCQ-24), and Social Support Rating Scale (SSRS). All scales that in Chinese, were standardized and suited to the Chinese population (see below for details).

#### Symptom checklist-90

The Symptom Checklist-90-Revised (SCL-90-R) is a 90-item self-report symptom inventory developed by Derogatis et al. (1976) in the mid-1970s and widely exploited to measure psychological status or screen for mental disorders. The SCL-90 consists of nine subscales with a total of 90 items: somatization, obsessive-compulsive, interpersonal sensitivity, hostility, phobic anxiety, paranoid ideation, psychoticism, anxiety, and depression. Each item is scored by Likert 5-point scale ranging from 1 (no symptoms), 2 (mild), 3 (moderate), 4 (serious), to 5 (severe), with a higher score representing a more evident degree of psychological distress symptoms. The SCL-90 has been proved to have acceptable psychometric properties for the screening of anxiety and depression among Chinese doctors (Dai et al., 2015). In addition, the standard (the mean of the sample plus a standard deviation) recommended by Dang et al. (2020) was acted as the positive threshold of anxiety and depression. In our study, the Cronbach’s α for depression and anxiety subscale was 0.89 and 0.88, respectively.

#### Psychological capital questionnaire

PsyCap was measured via the Psychological Capital Questionnaire (PCQ-24), which was developed by Luthans et al. (2007a) and the Chinese version has shown good reliability among Chinese physicians (Tian et al., 2020). The PCQ-24 consists of four dimensions (i.e., self-efficacy, hope, optimism, and resilience), with each dimension including six items. Each of the items is scored on a Likert 6-point scale, with 1 indicating potent disagreement and 6 indicating strong agreement. A higher score generally indicates a higher level of PsyCap and better mental health. In our study, the Cronbach’s α was 0.93 for the total scale, with 0.74, 0.88, 0.88, and 0.83 for the subscales of self-efficacy, hope, resilience, and optimism, respectively.

#### Social support rating scale

Social support rate scale (SSRS), compiled by Xiao, was adopted to measure individuals’ social support, including three dimensions: objective social support, subjective social support, and the availability of social support (Xiao, 1994). The SSA subscale contained three items, such as “How often you participate in activities organized by groups (party member organizations, trade unions, student unions, etc.).” Each item scoring 1–4 points, with a higher score representing better SSA. It has been demonstrated to be a reliable and valid measure for assessing SSA among Chinese physicians (Song et al., 2021). In our study, the Cronbach’s α was 0.64 for the subscale of SSA.

### Ethics approval

The study was reviewed and approved by the ethics committee at Xuzhou Medical University. All research methods were performed in accordance with relevant guidelines of Declaration of Helsinki. Completion of the surveys was deemed as an indication of participant consent.

### Statistical analysis

Descriptive analysis was used to describe and compare the socio-demographic data (e.g., gender, age, and working-years). For continuous variables, mean and standard deviation (SD) were used; and for discrete variables, rate (or proportion) was used. Pearson’s correlation analyses of the four variables (PsyCap, SSA, anxiety and depressive symptoms) were performed using SPSS version 21.0. AMOS 24.0 was employed to test the validity of scales. G*power 3.1.9.2 was used to calculate the sample size. To test the significance of the moderated mediation model in this study, we adopted model 4 and 59 of PROCESS v3.4 macro for SPSS provided by Hayes and Rockwood (2019). This approach is based on ordinary least-squares regression and the bootstrap method. A simple slope test was used to initially probe the moderating effect. The bootstrapped conditional effect was used to show the interactions and the Johnson–Neyman (J–N) technique was applied for probing interactions more specifically (Hayes, 2018). The J–N technique can identify regions in the range of the moderator variable where the effect of the antecedent variable on the outcome variable is statistically significant or not significant. The number of bootstrap confidence intervals was 5,000.

## Results

### Sample size

We adopted the G*power 3.1.9.2 to calculate the sample size. A total of 536 of 593 EPs submitted valid questionnaires, with the response rate of 90.4%. *Post hoc* analysis in G*power was employed to compute the achieved power (1-β) of the simple size of our study. Because the process macro was based on the multiple regression model, fixed model of linear multiple regression was set as the statistical test. The calculated parameters were as follows: effect size *f*^2^ was inputted as 0.15, α was 0.05, the total sample was 536, and the number of final predictors was 10. The result showed that a statistical power of 100% was achieved in the regression model, indicated that 536 EPs reached the necessary sample size.

### Common method bias

Harman’s single-factor test was used to probe common method bias. The result found that nine factors had eigenvalues greater than 1, and the first factor of the amount of variation explained was 29.38%, less than 40% of the critical criterion. Therefore, the common method bias in our study was not strong.

### Validity and reliability

To test the construct validity, we assessed for discriminant validity and convergent validity. The discriminant validity was measured by comparing the AVE square root of the scales with the correlation coefficient between the scale variable and other variables. If the AVE square root > the absolute value of correlation coefficient, it showed that the constructs had adequate discriminant validity (Fornell and Larcker, 1981). As shown in Table 1, the AVE square root of four scales were between 0.63 and 0.79, which higher than the absolute value of correlation coefficient except for the coefficient between anxiety and depression. Previous researches had demonstrated that anxiety and depression subscales of SCL-90 have greatly discriminant validity (Morgan et al., 1998; Bech et al., 2014). In general, the constructs had adequate discriminant validity in our study.

**TABLE 1:** Descriptive statistics and bivariate correlations.

	M ± SD	1	2	3	4
(1) Dep	1.56 ± 0.54	0.63			
(2) Anx	1.52 ± 0.54	0.88***	0.67		
(3) SSA	8.01 ± 2.00	-0.22***	-0.17***	0.63	
(4) PsyCap	100.44 ± 16.58	-0.43***	-0.39***	0.20***	0.79

M, mean; SD, standard deviation; Dep, depression; Anx, anxiety; SSA, social support availability; PsyCap, psychological capital. The diagonal is the AVE square root of the corresponding scale. Two-tailed test, ***p < 0.001.

The convergent validity was evaluated the magnitude of the standardized factor loadings (SFL) and composite reliability (CR). The SFL values of the measurement model of scales were between 0.572 and 0.913 except for only three items (0.446, 0.464, and 0.467), which were above the threshold of 0.5 in general (Hair et al., 2010). Furthermore, the CR values of four constructs ranged from 0.65 to 0.89, which all exceeded the convergent validity threshold of 0.6 (Hair et al., 2010). Hence, the convergent validity of scales was appropriate.

For reliability, we additionally reported the CR values besides the Cronbach’s α coefficient. The CR values for the scales ranged from 0.65 to 0.89, which agreed with Fornell–Larcker criteria of 0.6 (Fornell and Larcker, 1981). Therefore, the scales used in our study had acceptable reliability.

### Preliminary analyses

A total of 593 emergency physicians were recruited in this study, and 536 valid data were screened out according to the exclusion criteria (the effective response rate = 90.4%), including 330 (61.6%) males and 206 (38.4%) females. 54.9% of emergency physicians were between the ages of 26 and 35. 67.4% of samples have been working for less than 10 years. See Table 2 for more details.

**TABLE 2:** Descriptive statistics of emergency physicians.

Category	*N* (%)
**Total**	536(100%)
**Gender**	
Male	330(61.6%)
Female	206(38.4%)
**Age**	
≤25	46(8.6%)
26–35	294(54.9%)
36–45	142(26.5%)
≥46	54(10.0%)
**Work-yrs**	
≤10	361(67.4%)
11–20	114(21.3%)
21–30	52(9.7%)
≥31	9(1.6%)
**Edu-bac**	
≤Associate degree	10(1.9%)
Bachelor’s degree	219(40.9%)
≥Master’s degree	307(57.2%)
**Pro-pos**	
Resident or intern	254(47.4%)
Attending physician	163(30.4%)
Deputy chief physician	75(14.0%)
Chief physician	44(8.2%)

SD, standard deviation; Work-yrs, working-years; Edu-bac, educational background; Pro-pos, professional post.

According to the standard recommended by Dang, we used 2.1 (1.52/1.56 + 0.54) as the positive threshold for anxiety and depression subscales in the present study. We found that 16.6% (89/536) of EPs had reach the threshold of anxiety, and 16% (86/536) of EPs could been considered had depression. In addition, the proportion of the comorbidity of anxiety and depression among EPs is 12.13% (65/536) in the present study.

### Correlation analysis

Pearson’s correlation analysis revealed that significant correlations existed between these variables. Specifically, depression was negatively related to SSA and PsyCap (*r* = –0.22 and –0.43, *p* < 0.001, respectively). Anxiety was also negatively associated with SSA and PsyCap (*r* = –0.17 and –0.39, *p* < 0.001, respectively). In addition, a positive correlation between PsyCap and SSA was identified (*r* = 0.20, *p* < 0.001). Moreover, the four components of PsyCap are all negatively related to anxiety and depression (Table 1).

### Mediation analyses

As shown in Table 3, anxiety had a negative impact on SSA (β = –0.17, *p* < 0.001). With the statistically significant effect of SSA on depression (β = –0.08, *p* < 0.001), it appears that anxiety had a positive direct effect (β = 0.86, *p* < 0.001), and further had an indirect effect on depression via the partially mediating role of SSA (indirect effect = 0.013, BootSE = 0.005, 95%BootCI [0.005, 0.023]) (Table 4). Hence, hypothesis 1 and 2 was supported.

**TABLE 3:** Model characteristics for moderated mediation analysis.

	Model 1 (Y = SSA)	Model 2 (Y = Depression)	Model 3 (Y = SSA)	Model 4 (Y = Depression)
Gender	0.20*	0.07	0.18*	0.07
Age	-0.20*	-0.02	-0.25*	-0.01
Work-yrs	0.03	0.02	0.06	0.01
Edu-bac	0.29***	-0.01	0.32***	-0.03
Pro-pos	0.08	-0.01	0.59	0.01
Anxiety (X)	-0.17***	0.86***	-0.11*	0.82***
SSA (M)		-0.08***		-0.07**
PsyCap (Z)			0.18***	-0.10***
X × Z			-0.02	-0.01
M × Z				0.06**
***R* ^2^**	0.07	0.77	0.10	0.78
***F***	6.50***	256.09***	7.05***	190.12***

SSA, social support availability; PsyCap, psychological capital; Work-yrs, working-years; Edu-bac, educational background; Pro-pos, professional post; X, independent variable; M, mediating variable; Z, moderating variable; Y, outcome variable. X, M, Z, and Y were all standardized. Two-tailed test, *p < 0.05, **p < 0.01, ***p < 0.001.

**TABLE 4:** Bootstrapped direct and indirect effects of mediation analysis.

*X*	Effect	BootSE	95%BootCI	
			LL	UL
Direct	0.861	0.021	0.819	0.902
Indirect	0.013	0.005	0.005	0.023

### Moderated mediation analyses

In the moderation analysis, the interaction of PsyCap and anxiety was insignificant (β = –0.01, *p* = –0.115), which displayed that PsyCap had no moderating effect in the relationship between anxiety and depression. Meanwhile, the interact term of anxiety and SSA was also insignificant (β = –0.02, *p* = 0.569), whereas the interaction of SSA and PsyCap was statistically significant (β = 0.06, *p* = 0.004) (Table 3). This indicated that PsyCap played a moderating role in the link between SSA and depression.

**FIGURE 2:**
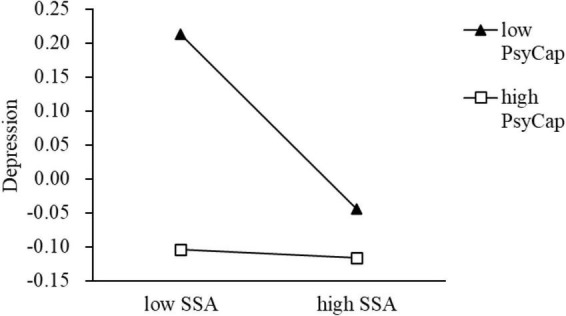
The simple slope test of the moderating effect of PsyCap on the relationship between SSA and depression. SSA, social support availability; PsyCap, psychological capital.

**FIGURE 3:**
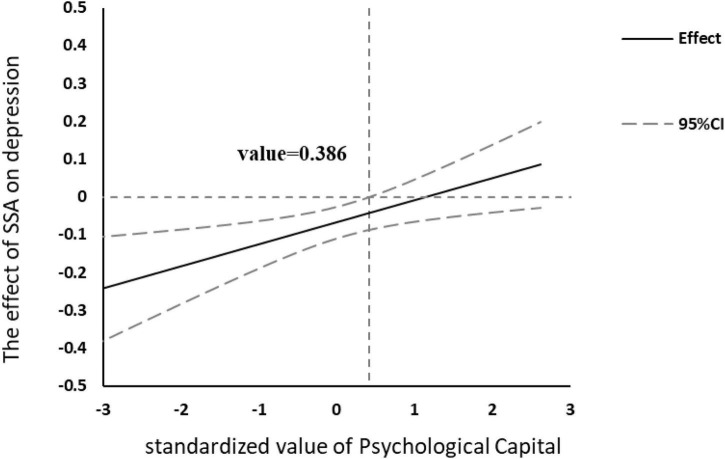
Effect of social support availability (SSA) on depression at different levels of psychological capital (PsyCap). The moderating effect of PsyCap was existed when the value of PsyCap ≤ 0.386.

In term of probing the moderation, when PsyCap was divided into high and low groups by M ± 1 SD, the simple slope test indicated that compared with EPs in high groups, the effect of SSA on depression was more pronounced among the low group (Figure 2). The analysis of conditional indirect effect also confirmed that the indirect effect was not significant when PsyCap was in the high group (Table 5). Furthermore, the J–N technique indicated that the standardized score of 0.386 on the PsyCap can be regarded as a point of transition between the statistically significant effect and the non-significant effect of SSA on depression (Figure 3). In addition, we also explored the moderating role of four components of PsyCap in the model. Results showed that self-efficacy (β = 0.07, *p* = 0.004), hope (β = 0.06, *p* = 0.010), and resiliency (β = 0.05, *p* = 0.015) had significant moderating effect in the link from SSA to depression except for optimism (β = 0.03, *p* = 0.143, Table 6). In all, hypothesis 3 was partially supported.

**TABLE 5:** Bootstrapped conditional direct and indirect effects.

Anxiety	PsyCap	Effect	BootSE	95%BootCI	
				LL	UL
Direct	Low	0.821	0.024	0.774	0.869
	High	0.817	0.037	0.744	0.890
Indirect	Low	0.012	0.006	0.001	0.025
	High	0.001	0.003	-0.007	0.008

The outcome variable: depression. The values of moderator: low = –1, high = 1.

**TABLE 6:** Model coefficients for moderated mediation analysis of components of PsyCap.

Predictors	Moderator = Self-efficacy		Moderator = Hope		Moderator = Resiliency	
	Model 1 (Y = SSA)	Model 2 (Y = Depression)	Model 3 (Y = SSA)	Model 4 (Y = Depression)	Model 5 (Y = SSA)	Model 6 (Y = Depression)
Gender	0.17	0.07	0.18	0.07	0.18	0.07
Age	-0.12	-0.04	-0.13	-0.03	-0.13	-0.04
Work-yrs	0.05	-0.01	0.05	0.01	0.05	-0.01
Edu-bac	0.24***	-0.01	0.23**	0.01	0.23**	0.02
Pro-pos	0.01	0.01	0.01	0.01	0.01	0.01
Anxiety (X)	-0.15**	0.87***	-0.14**	0.83***	-0.16**	0.82***
SSA (M)		-0.08***		-0.08***		-0.08***
Moderator (Z)	0.10*	-0.01	0.11*	-0.07**	0.08	-0.07**
X × Z	-0.01	0.07**	-0.02	-0.01	-0.03	-0.01
M × Z		0.07**		0.06*		0.05*
***R* ^2^**	0.07	0.78	0.07	0.78	0.07	0.78
***F***	4.50***	168.70***	4.62***	169.40***	4.29***	169.16***

SSA, social support availability; Work-yrs, working-years; Edu-bac, educational background; Pro-pos, professional post; X, independent variable; M, mediating variable; Z, moderating variable; Y, outcome variable. X, M, Z, and Y were all standardized. Insignificant results were not shown. Two-tailed test, *p < 0.05, **p < 0.01, ***p < 0.001.

## Discussion

This cross-sectional survey was conducted to find the psychological symptoms (anxiety and depressive symptoms), SSA and PsyCap level of EPs in China. And to date, this investigation was the first study to explore the moderation mediation mechanism of SSA and PsyCap in the link from anxiety to depression (SSA as the mediating variable and PsyCap as the moderating variable). Interestingly, in our study the positive rates for anxiety and depression were almost the same (16%) and the proportion that met the positive criteria for both symptoms was 12.13%. The regression model also showed anxiety had a strong positive association with depression. This result reaffirmed the high comorbidity of anxiety and depression at the cross-sectional level, which was consent with the conclusion of large cohort study (Lamers et al., 2011).

Central to our research findings is the examination in which SSA and PsyCap are postulated as intervening variables in the relationship between anxiety and depression in a model. Our findings demonstrated that the mediation effect of SSA in the pathway from anxiety to depression and the moderating role of PsyCap in the link between SSA and depression were both significant. The findings also provided new insights into the detail of how PsyCap can play a significant moderating role. Specifically, it was found that the moderating effect PsyCap on the link between SSA and depression was significant only when PsyCap was at the low level.

In our study, SSA played a mediating role in the link from anxiety to depression, which verified the subjective cognitive appraisal of social support (Cohen and McKay, 2020). When an individual’s evaluation of an event tends to be negative, the negative belief about self will occur, which is central to the development of depression (Otani et al., 2018). High level of SSA can make individuals have less negative appraisal of stressful events (Lakey and Cohen, 2000). The specificity of the occupation requests the EPs to be fully prepared for unpredictable emergency incidents, which brings them frequently negative emotions or behaviors by the patients from different types of families, especially those who are drunk, irritable, aggressive or even violent (Liu et al., 2015). These factors have been proven to be risk factors for depression among EPs in China (Chen et al., 2022). When EPs are confronted with these stressful anxiety events, the appraisal system is activated. If they are proactive and have resources available for support that are sufficient to deal with these things, that is, high levels of SSA, then the level of depression will be reduced and. Relatively, if EPs don’t seek support from friends and family in a timely and active manner, that is, the level of SSA is low, they wrongly believe subjectively that no one helps them, amplifying the negative appraisals of stressful events and deteriorating the anxiety symptoms into depression (Hart and Hittner, 1991; Starr et al., 2014).

Through the moderation analysis, we found that the significant dynamic effect of PsyCap in the relationship between SSA and depression. Compared to EPs with high PsyCap, the alleviating effect of SSA on depression was more significant. Specifically, when the amount of PsyCap was below a certain value (varied according to the sample specific value), the effect of SSA on alleviating depression became more pronounced with the adding PsyCap score. But when the PsyCap score reached a critical value, the moderating effect was non-significant. The possible interpretation for this may be twofold. First, people with low level of PsyCap are not very good at perceiving and utilizing social support. They cannot make good use of the resources of social support around when suffering from stress. Second, high PsyCap is associated with low depression (Bakker et al., 2017), and the promoting interaction between PsyCap and social support (Liu et al., 2013) makes individuals with high psychological capital have no great improvement in the utilization of social support. Therefore, for EPs with high PsyCap, if depression symptoms occur, they need to find other psychological resources for relief.

Based on the moderation analysis of overall PsyCap, we further conducted the same model to explore the moderating effect of four components of PsyCap. The results indicated that self-efficacy, hope and resiliency exerted the identical dynamic effect as PsyCap, that was these traits could enhance SSA in alleviating depressive symptoms. This corroborated previous research and further explored the interaction between SSA and these psychological traits. Holahan and Holahan (1987) found self-efficacy could function indirectly through its effect on social support in preventing depression by a longitudinal study. A recent study also showed that self-efficacy and social support could alleviate depression and improve psychological well-being among Chinese nurses (Xie et al., 2020). The growth of low level of self-efficacy can make individuals better use of the social support around them and believe in their ability to regard demands as challenges rather than treats (Weber et al., 2013). Similarly, a study (Tao et al., 2022) found that hope protected individuals from depression by enhancing social support (including the SSA dimension). Another study (Zhao et al., 2018) showed that resiliency moderated the relationship between social support and depressive symptoms. These traits can help EPs discover the positive connotation behind stressful events and fully tap their supports to cope with these difficulties (Feng and Yin, 2021). Unexpectedly, optimism did not have the moderating effect in our study. We inferred that this may be due to the cultural differences in optimism. The effect of optimism on individuals’ behavior differed by nationality and culture (Wang et al., 2021a). In general, due to the influence of Confucian culture, Chinese had lower level of optimism than western group (Moyer et al., 2009). Coupled with overwhelming workload, optimism may have little effect on SSA of EPs.

Our study is not free from limitations. First, due to the cross-sectional observational nature of our study, the time causality between these factors cannot be robustly determined and established. We can only try to explore the underlying mechanisms. Second, we did not examine the precedence of anxiety and depression and the bidirectional causal relationship between anxiety and depression may be more complicated. Confounding factors are not the only ones included in our study, such as genetic, socio-environmental stressors, etc. Third, the subscale that was used to assess SSA was not custom-designed. It is necessary to develop a specialized measurement scale in the further researches. Fourth, our sample only focus on the EPs of the top-level hospital in Jiangsu province. There is a need for further studies to authenticate whether our conclusion can be generalized to other medical practitioners in other grade hospitals or other Chinese provinces with different profiles.

### Theoretical and practical implications

The value of the present study was to verify the subjective cognitive appraisal theory of SSA and the dynamic effect of PsyCap in relieving symptoms of anxiety and depression among EPs in China. First, our study demonstrated the high co-existence of anxiety and depression at the data level. In clinical diagnosis, the two are generally not diagnosed separately (Ter Meulen et al., 2021). Therefore, mental health needs close attention among EPs, especially for anxiety and depressive symptoms. Second, the present study found that SSA mediated the conduction pathway from anxiety to depression, which verified the appraisal mechanism of social support for individual mental health protection. Specifically, SSA could reduce the effect of anxiety in depression among EPs. This reminds EPs that when they experience psychological symptoms, such as anxiety, they need to actively seek support around them in time and improve SSA to buffer the negative impact of psychological stress. Third, our study displayed that PsyCap played a moderating role in the link between SSA and depression. More importantly, we found that the moderating effect of PsyCap only works in the low level. Therefore, psychological capital intervention can be applied to enhance SSA to further alleviate anxiety and depressive symptoms. Hospitals should organize activities to improve PsyCap of EPs with low PsyCap, which focus on self-efficacy, hope and resiliency. EPs with high PsyCap need to keep the *status quo*, make their anxiety and depression at a low level, and ensure their mental health. According to the ‘Healthy China 2030’ program, EPs should be more likely to have priority over others to be screened for depression to prevent mental health problems. With their mental health improved, they can provide better medical care services.

## Conclusion

Our study explored the mediating role of SSA and the moderation effect of PsyCap in the relationship between anxiety and depression. The results found that anxiety had a strong association with depression and SSA mediated the pathway between the two. Furthermore, PsyCap moderated the second half of the mediated pathway (the link from SSA to depression). More specifically, the alleviating effect of PsyCap was more pronounced at low level of PsyCap.

## Data availability statement

The original contributions presented in this study are included in the article/supplementary material, further inquiries can be directed to the corresponding author.

## Author contributions

XL and HX developed the rationale for the study. HX and LP analyzed and interpreted data as well as wrote up the initial draft of the manuscript. HX and ZW revised subsequent manuscripts. All authors read and approved the final manuscript.
